# Maternal glucose homeostasis is impaired in mouse models of gestational cholestasis

**DOI:** 10.1038/s41598-020-67968-6

**Published:** 2020-07-13

**Authors:** Elena Bellafante, Saraid McIlvride, Vanya Nikolova, Hei Man Fan, Luiza Borges Manna, Jenny Chambers, Mavis Machirori, Anita Banerjee, Kevin Murphy, Marcus Martineau, Kristina Schoonjans, Hanns-Ulrich Marschall, Peter Jones, Catherine Williamson

**Affiliations:** 1 School of Life Course Sciences, King’s College London, London, UK; 2 Women’s Health Research Centre, Imperial College London, London, UK; 3 Guy’s and St. Thomas’ NHS Foundation Trust, London, UK; 4 Department of Medicine, Imperial College London, London, UK; 5 Department of Surgery and Cancer, Imperial College London, London, UK; 6 Laboratory of Integrative and Systems Physiology, École Polytechnique Fédérale de Lausanne, Lausanne, Switzerland; 7 Department of Molecular and Clinical Medicine, Sahlgrenska Academy, University of Gothenburg, Gothenburg, Sweden; 8 Maternal and Fetal Disease Group, Hodgkin Building, Guy’s Campus, King’s College London, London, SE1 1UL UK

**Keywords:** Physiology, Metabolic disorders

## Abstract

Women with intrahepatic cholestasis of pregnancy (ICP), a disorder characterised by raised serum bile acids, are at increased risk of developing gestational diabetes mellitus and have impaired glucose tolerance whilst cholestatic. FXR and TGR5 are modulators of glucose metabolism, and FXR activity is reduced in normal pregnancy, and further in ICP. We aimed to investigate the role of raised serum bile acids, FXR and TGR5 in gestational glucose metabolism using mouse models. Cholic acid feeding resulted in reduced pancreatic β-cell proliferation and increased apoptosis in pregnancy, without altering insulin sensitivity, suggesting that raised bile acids affect β-cell mass but are insufficient to impair glucose tolerance. Conversely, pregnant *Fxr*^*−/−*^ and *Tgr5*^*−/−*^ mice are glucose intolerant and have reduced insulin secretion in response to glucose challenge, and *Fxr*^*−/−*^ mice are also insulin resistant. Furthermore, fecal bile acids are reduced in pregnant *Fxr*^*−/−*^ mice. Lithocholic acid and deoxycholic acid, the principal ligands for TGR5, are decreased in particular. Therefore, we propose that raised serum bile acids and reduced FXR and TGR5 activity contribute to the altered glucose metabolism observed in ICP.

## Introduction

Intrahepatic cholestasis of pregnancy (ICP) is a liver disorder which affects approximately 1 in 140 pregnancies in the UK^1^. It is characterised by the presence of pruritus and abnormal liver function tests with elevated serum bile acid concentrations, appearing usually during the third trimester of pregnancy and persisting until delivery. ICP is associated with adverse pregnancy outcomes, including increased rates of spontaneous preterm labour, fetal distress, prolonged neonatal unit admission, and intrauterine death^2–4^. The 16-year-old children of women with ICP are more likely to have subsequent obesity and dyslipidaemia^5^. Women with ICP have an increased risk of developing gestational diabetes mellitus (GDM) and have significant biochemical and endocrine changes such as increased basal endogenous glucose production, reduced glucagon-like peptide-1 (GLP-1) secretion and decreased insulin sensitivity, that lead to impaired carbohydrate metabolism while they are cholestatic^6–9^.

Bile acid metabolism and synthesis is primarily regulated by the intestinal and hepatic farnesoid X receptor (FXR)^10^. Briefly, in the liver, activation of FXR by bile acids upregulates the expression of small heterodimer partner (SHP), which leads to repression of CYP8B1, and to a lesser extent, CYP7A1, key enzymes in bile acid synthesis^11,12^. Furthermore, expression of transporters involved in bile acid uptake are downregulated (NTCP and OATP1B1/3 (OATP1B2 in mice)) and canalicular export of bile acids is increased (through induction of BSEP and MRP2)^13^.In the intestine, FXR induces expression of fibroblast growth factor 19 (FGF19 or FGF15 in mice), which feeds back to the liver where it has a critical role in repression of CYP7A1 and CYP8B1 gene expression^11,12,14^. As well as bile acid metabolism, FXR has also been implicated in the regulation of glucose homeostasis^15^. Bile acid administration to diabetic *db/db* mice decreases hepatic gluconeogenesis and lowers plasma glucose levels^16^. Moreover, murine *Fxr* deficiency results in insulin resistance and impaired glucose tolerance, whereas its activation improves insulin sensitivity^16^. The expression of several islet-specific genes is altered and insulin secretion is also impaired in mice lacking *Fxr*^17^. FXR activation stimulates insulin secretion in mouse β-cells by inhibiting ATP-sensitive potassium channel activity^18^ and modulates gluconeogenesis and insulin sensitivity in liver, muscle, and adipose tissue^19^. In humans, the increase in serum bile acids in response to an oral glucose tolerance test is reduced in prediabetic and severely obese patients; and in type 2 diabetes patients, bile volume and composition are changed^20^. Increased serum concentrations of several bile acid species has also been observed in women with GDM, compared to women with uncomplicated pregnancies^21^. Very little is known about the relationship between gestational signals that influence bile acid and glucose homeostasis or β-cell adaptations in uncomplicated pregnancy compared with gestational cholestasis. Hepatic FXR function is reduced in normal pregnancy as a consequence of raised concentrations of reproductive hormones, including progesterone sulphates^22^ and 17β‐estradiol^23^, and levels of progesterone sulphates are even higher in cholestatic pregnancies^24^, suggesting that gestational alterations in FXR signalling may contribute to susceptibility to GDM in ICP.

The membrane bile acid receptor TGR5 (*GPBAR1*) has also been implicated in glucose metabolism. In the small intestine, activation of TGR5 by bile acids leads to GLP-1 release^25^. Following release into the circulation, GLP-1 binds to its cognate receptors on pancreatic β-cells, stimulating the potentiation of glucose-stimulated insulin release and insulin gene transcription^26^. Moreover, TGR5 is able to induce insulin secretion in murine pancreatic islets^27^. Activation of TGR5 by synthetic agonists has been shown to reduce plasma glucose levels in rodents^25,28^. Given that normal pregnancy is associated with reduced FXR function^29–31^, thought to be further exacerbated in ICP^22^, it is likely that altered concentrations of specific bile acids reach the luminal compartment of the intestine to activate both FXR and TGR5. Furthermore, we have previously demonstrated that progesterone metabolites which are raised in pregnancy and ICP can influence TGR5 signalling^24^. In light of the involvement of both receptors in glucose metabolism, we hypothesised that reduced activity of FXR and TGR5 may have a role in aberrant glucose homeostasis in ICP and increased susceptibility to GDM.

## Research design and methods

### Animal studies

Animal studies were carried out at King’s College London in accordance with the Animals (Scientific Procedures) Act 1986 Amendment Regulations 2012 and approved by the King’s College London Animal Welfare and Ethical Review Body and the Home Office. Mice were maintained on a standard diet (CRM; Special Diets Services, UK), allowed free access to food and water and housed in a temperature- and light-controlled environment (12 h light/dark cycle). Sample sizes (6–8 animals per group) were based on previously published studies from our group involving similar analyses. Mice were randomly assigned to treatment groups. Blinding was not possible for in vivo experiments.

7–8 week old C57BL/6J mice were purchased from Envigo, UK. *Fxr*^*−/−*^ and *Tgr5*^*−/−*^mice (maintained on a C57BL/6J background) were generated and validated in the laboratory of Dr. Kristina Schoonjans, Lausanne, Switzerland, and have been previously described in detail^25,32^. C57BL/6J mice were used as controls unless otherwise stated. Female mice were paired with male C57BL/6J mice and the day of identification of a copulatory plug was considered to be day 1 of pregnancy (D1). Age-matched virgin mice were used as non-pregnant controls (D0). Bromodeoxyuridine (BrdU) was administered via drinking water (1 mg/ml) for 8 days prior to euthanisation. Females were euthanised on D15 or D18 of pregnancy, or equivalent for non-pregnant controls, by CO_2_ inhalation after 4 h of food deprivation.

To induce hypercholanaemia, C57BL/6J mice were fed CRM diet supplemented with 0.5% cholic acid (CA; Special Diets Services, UK) from D1 of pregnancy until euthanisation by CO_2_ inhalation at D15 or D18, as previously described^5^. Non-pregnant controls were fed CA-supplemented diet for the equivalent time period.

## Glucose and insulin tolerance tests

Glucose tolerance tests (GTT) and insulin tolerance tests (ITT) were performed in mice at D18 of pregnancy, or equivalent for non-pregnant controls, as described previously^33^. Mice were fasted from 9am for 6 h and administered either 2 g/kg body weight of glucose or 0.75 IU/kg of insulin intraperitoneally, unless otherwise stated. Blood glucose concentrations were measured from the tail vein using a FreeStyle Lite glucometer (Abbott Diabetes Care, UK). Blood samples were also collected from the tail vein during GTT and plasma insulin measured by insulin ELISA (Millipore, UK), according to manufacturer’s instructions.

## Haematoxylin and eosin (H&E) staining

Pancreata were fixed overnight in 10% neutral buffered formalin, washed and embedded in paraffin. 5 µm-thick sections were cut and mounted onto poly-L-lysine coated slides, as previously described^34^. Slides were stained with H&E and imaged using an Eclipse TE200-U fluorescent microscope (Nikon, Japan) and Metafluor PC software. Slides were viewed at 10 × magnification.

## Immunohistochemistry and islet morphology

Pancreas samples were processed as described above. Slides underwent antigen retrieval using citrate-based antigen unmasking solution (Vector Laboratories, UK). Sections were co-incubated with guinea pig anti-insulin (1:200; Dako, UK) and mouse anti-BrdU (1:10; BrdU labelling and detection kit I, Roche, UK). Sections were then incubated with secondary antibodies: goat anti-guinea pig IgG (1:50; Thermo Fisher Scientific, UK) and sheep anti-mouse Ig-fluorescein (1:10; Roche, UK). Slides were mounted using Fluoroshield mounting medium (Sigma-Alrich, UK).

Slides were imaged as above, at 20 × magnification. Total β-cell nuclei and BrdU-positive nuclei within an islet were counted manually, and total area of insulin-positive staining measured using ImageJ software (imagej.nih.gov).

## TUNEL assays

Pancreas samples were processed as described above. Cell apoptosis was quantified by Terminal deoxynucleotidyl transferase (TdT) dUTP Nick-End Labeling (TUNEL) assay (Roche, UK), according to manufacturer’s protocol. Slides were imaged as above, at 20 × magnification.

## mRNA expression

Total RNA was extracted from tissues using Qiazol lysis reagent (Qiagen, UK) according to manufacturer’s instructions with the modification of precipitation in isopropanol at − 80 °C. RNA was treated with DNase (Qiagen, UK) and reverse transcribed using High-Capacity cDNA Reverse Transcription Kit (ThermoFisher Scientific, UK). Real-time quantitative PCR was performed with a Viia7 system (ThermoFisher Scientific, UK), using Sybrgreen (Sigma-Aldrich, UK). The fold change in gene expression is given as 2^−∆∆Ct^.

## Western blotting

Total and phosphorylated Akt were measured in liver samples by Western blotting. Samples were separated using a 12% SDS–polyacrylamide gel and transferred onto a PVDF membrane, as previously described^35^. The membrane was probed for total Akt (1:1,000; Cat no. 4685, Cell Signaling Technology, USA) and Phospho-Akt (Ser473; 1:1,000; Cat no. 4060, Cell Signaling Technology, USA). GAPDH was used as a loading control (MAB374; 1:4,000; Millipore, USA). Proteins were detected using chemiluminescence (Millipore, USA).

## Fecal bile acid measurements

Feces were removed from the colon and frozen immediately upon euthanisation of the mice by CO_2_ inhalation. Fecal bile acid concentrations were measured using ultra-performance liquid chromatography tandem mass spectrometry, as previously described^36^. Briefly, approximately 50 mg feces was dissolved in 500 µl methanol containing 2.5 µl deuterated internal standards and homogenised thoroughly. Samples were vortexed, centrifuged, and the supernatant was evaporated and reconstituted in 200 µl of methanol:water (1:1). Bile acids were separated using gradient elution on a Kinetex C18 column (Phenomenex, USA). Signal was detected using a QTRAP 5500 mass spectrometer (Sciex, Canada) with multiple reaction monitoring in negative mode, and bile acids were quantified using external standard curves. Bile acid concentration is given per mg dry weight of feces.

### Statistical analysis

Data are expressed as mean ± standard error of the mean (SEM), unless otherwise stated. Statistical analysis was carried out using GraphPad Prism 7 software (GraphPad Software, USA). Data were evaluated for normality using the Shapiro–Wilk normality test and analysed by one-way ANOVA followed by Newman–Keuls post-hoc analysis, unless otherwise specified.

## Results

### Glucose tolerance and insulin sensitivity is impaired in control and cholic acid-fed pregnant mice

**Figure 1:**
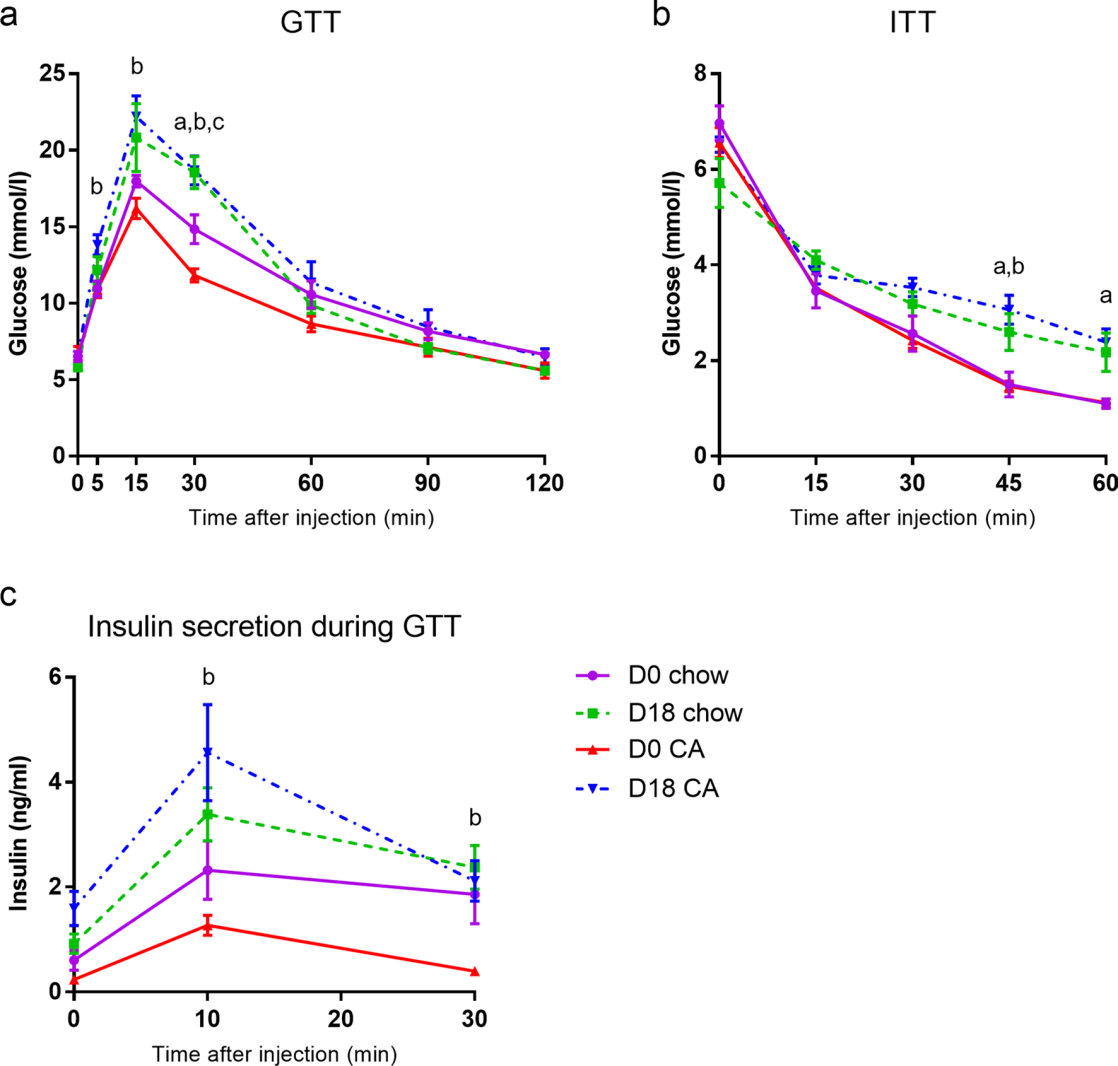
Glucose tolerance and insulin sensitivity is impaired in control and cholic acid-fed pregnant mice. Mice were fed cholic acid (CA)-supplemented diet from D1 of pregnancy until euthanisation at D18 (or equivalent for non-pregnant controls). **a** Glucose tolerance tests (GTT) were performed at D18 (or equivalent for non-pregnant controls). D0 chow, n = 9; D18 chow, n = 6; D0 CA, n = 8; D18 CA, n = 6. **b** Insulin tolerance tests (ITT). D0 chow, n = 6; D18 chow, n = 7; D0 CA, n = 6; D18 CA, n = 6. **c** Plasma insulin concentrations after glucose challenge. n = 6 for all groups. *p* < 0.05 as determined by repeated-measures two-way ANOVA followed by Bonferroni’s post-hoc test: ^a^D0 chow v D18 chow, ^b^D0 CA v D18 CA, ^c^D0 chow v D0 CA.

To investigate how hypercholanaemia may affect insulin secretion and glucose homeostasis, as well as pancreatic islet physiology and β-cell function during gestation, wild type pregnant mice were challenged with a diet supplemented with 0.5% cholic acid (CA), the principal bile acid that is raised in ICP, which we have previously shown increases serum bile acid levels^32^. Glucose tolerance tests showed that pregnant mice were mildly glucose intolerant when compared to their diet-matched non-pregnant controls. However, there was no difference in glucose tolerance between the pregnant groups (Fig. 1a). Consistent with this, pregnant mice fed with chow or CA diet both had impaired insulin sensitivity when compared to their non-pregnant controls (Fig. 1b), but there was no difference in insulin resistance between pregnant groups. To test if bile acid overload also affects insulin secretion in mice, insulin levels were assessed at different time points during the glucose challenge. While CA-fed pregnant mice showed significantly increased insulin secretion compared to non-pregnant CA-fed mice, there was no difference between any other groups (Fig. 1c).

## Hypercholanaemia induced by CA feeding reduces islet expansion during pregnancy

**Figure 2:**
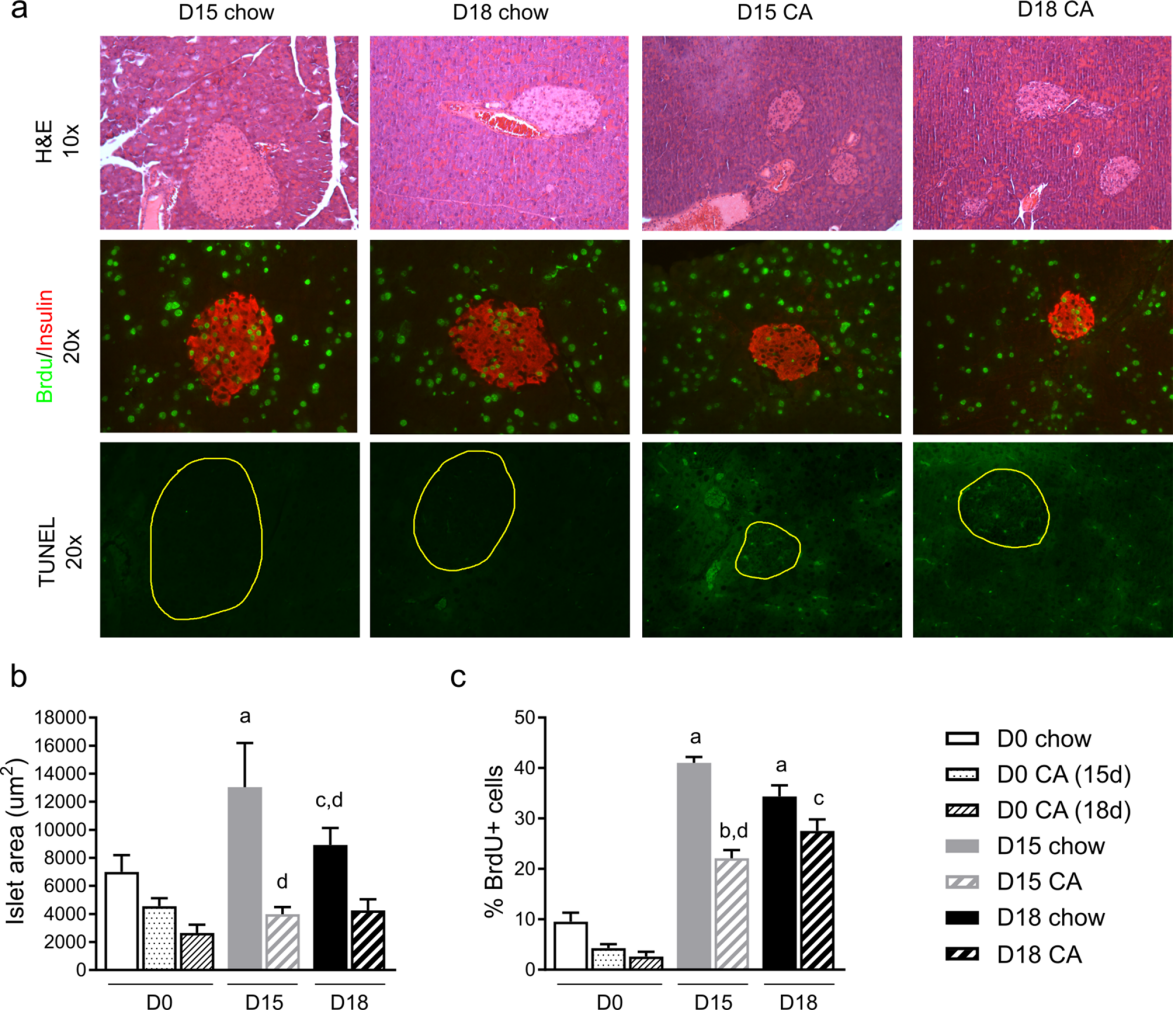
Hypercholanaemia reduces islet expansion during pregnancy. Mice were fed a chow diet supplemented with cholic acid (CA) from D1 of pregnancy until euthanisation at D15 or D18 (or equivalent for non-pregnant controls). **a** Representative islets showing H&E staining, BrdU (green) and insulin (red), and TUNEL (green; islet outlined in yellow) immunostaining from pregnant mice. **b** Islet area was calculated using ImageJ software. n = 4 mice per group, total islet number 17–31. **c** Percentage of BrdU positive cells in islets. 9–35 islets analysed per group. *p* < 0.05 for the following comparisons: ^a^vs D0 chow, ^b^vs D0 CA (15d), ^c^vs D0 CA (18d), ^d^vs D15 chow.

H&E and BrdU staining demonstrated that islet area reached its peak at D15 in control mice, consistent with the published literature^37^, decreasing again at D18 (Fig. 2a, b). In comparison, islet area was decreased in CA-fed dams at both gestational day 15 (D15) and D18 (Fig. 2a, b). In line with this, BrdU positive cells represent over 40% of total β-cell number at D15 in pregnant control mice, while in CA-fed females proliferating β-cells constitute less than 30% both at D15 and D18 (Fig. 2c). TUNEL assays performed on pancreas sections revealed that islets from both non-pregnant and pregnant mice fed with chow diet do not show any apoptotic cells, while cholestatic females showed increased apoptosis, especially during pregnancy (Fig. 2a). In vitro studies demonstrate that bile acids can enhance glucose-stimulated insulin secretion in mouse islets (Supplementary Fig. S1), confirming previous reports^18,38^.

## *Fxr* deficiency induces insulin resistance during pregnancy

**Figure 3:**
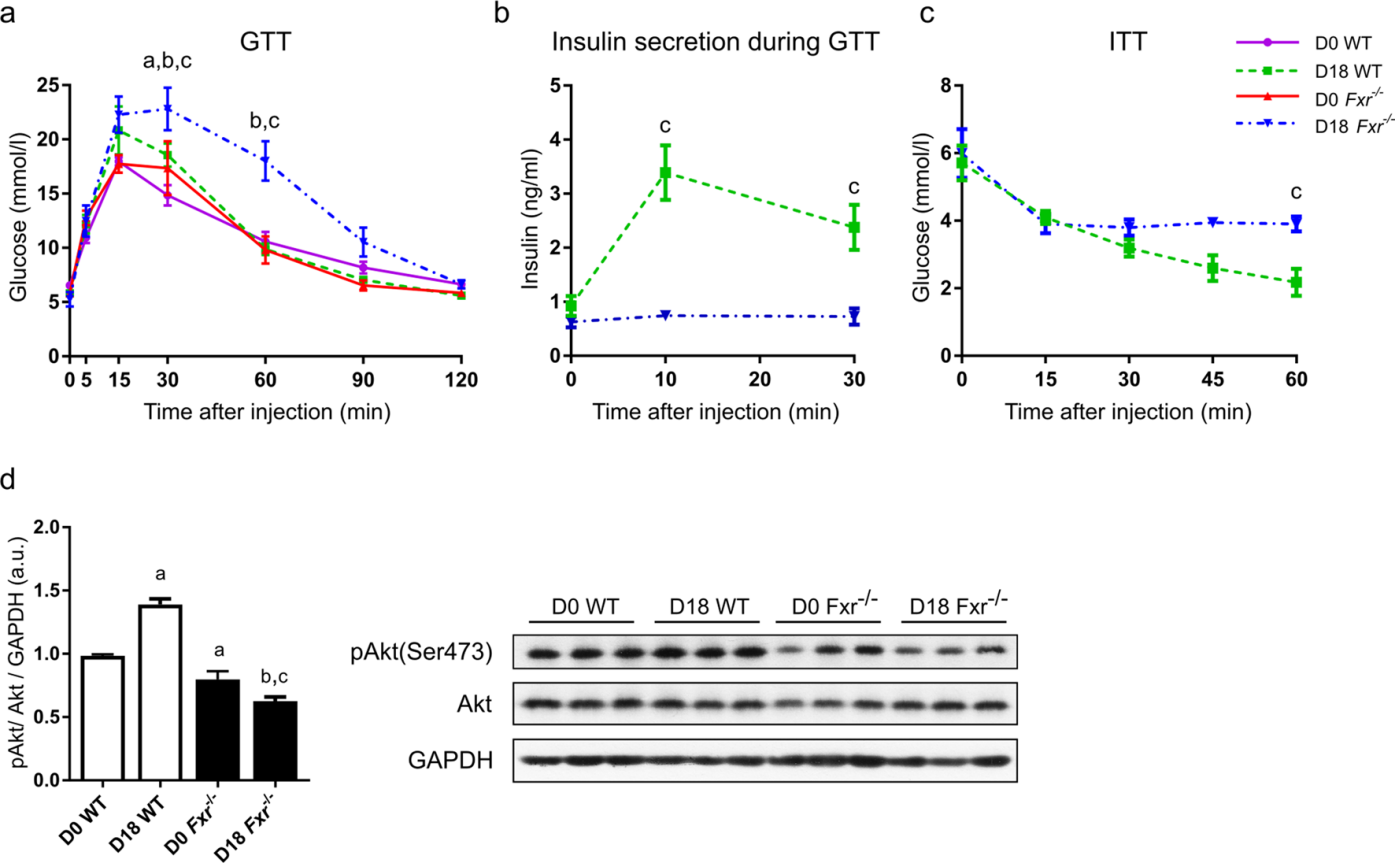
*Fxr* deficiency during pregnancy causes glucose intolerance and insulin resistance. Glucose metabolism was assessed in non-pregnant (D0) and pregnant (D18) wild type (WT) and *Fxr*^*−/−*^ mice at gestational day 18 (or equivalent in D0 controls). **a** Glucose tolerance tests (GTT) were performed. D0 WT, n = 8; D18 WT, n = 6; D0 *Fxr*^*−/−*^, n = 3; D18 *Fxr*^*−/−*^, n = 5. **b** Plasma insulin concentrations after glucose challenge in D18 mice. D18 WT, n = 6; D18 *Fxr*^*−/−*^, n = 5. **c** Insulin tolerance tests were performed on D18 mice. D18 WT, n = 8; D18 *Fxr*^*−/−*^, n = 5. p < 0.05 as determined by repeated-measures two-way ANOVA followed by Bonferroni’s post-hoc test, for the following comparisons: ^a^ D0 WT vs D18 WT, ^b^ D0 *Fxr*^*−/−*^ vs D18 *Fxr*^*−/−*^, ^c^ D18 WT vs D18 *Fxr*^*−/−*^. **d** Western blot analysis of hepatic phosphorylated Akt protein at residue Ser-473 and total Akt protein. Full-length blots are presented in Supplementary Fig. S2. n = 3 per group. *p* < 0.01 for the following comparisons: ^a^vs D0 WT, ^b^vs D18 WT, ^c^ vs D0 *Fxr*^*−/−*^.

To enable the investigation of the impact of suppressed FXR activity on susceptibility to GDM, glucose metabolism was also studied using pregnant *Fxr*^*−/−*^ mice. In contrast to the CA diet model, glucose tolerance tests showed that D18 *Fxr*^*−/−*^ mice are significantly glucose intolerant compared to both D0 *Fxr*^*−/−*^ and the D18 control mice presented in Fig. 1 (Fig. 3a). This is accompanied by a marked blunting of the insulin response to the glucose challenge in these mice (Fig. 3b). D18 *Fxr*^*−/−*^ mice also displayed significant insulin resistance compared to their control counterparts (Fig. 3c). Phosphorylation of Akt was also significantly reduced in the liver of D0, and even more so in D18, *Fxr*^*−/−*^ mice (Fig. 3d). In contrast to CA-fed mice, analysis of BrdU staining did not reveal any differences in β-cell proliferation or islet area between wild type and *Fxr*^*−/−*^ mice (Supplementary Table S1, S2). Overall, deficiency of *Fxr* results in insulin resistance and diminished insulin secretion in pregnancy.

## *Fxr* deficiency is associated with a decrease in fecal bile acids

**Figure 4:**
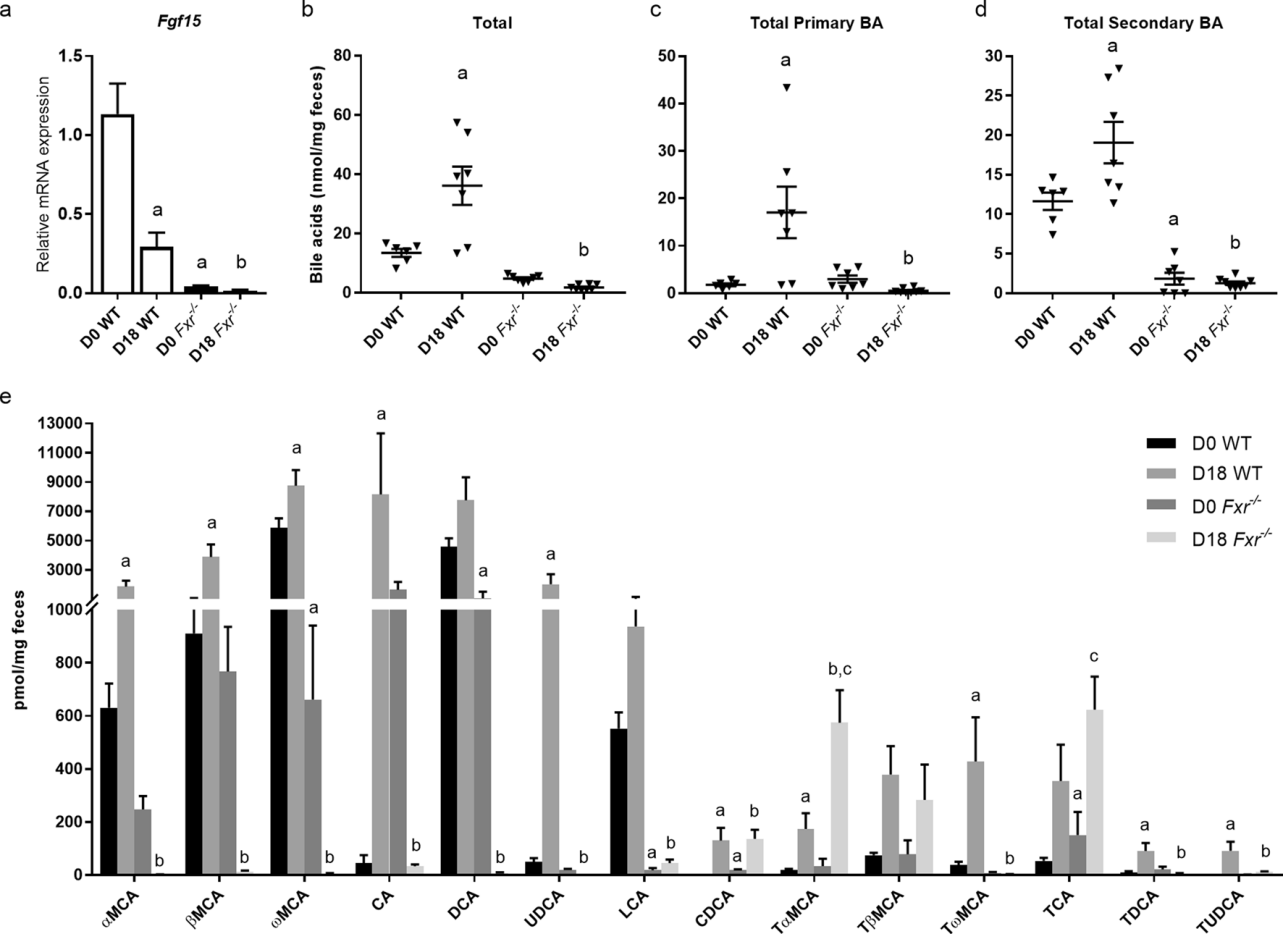
Fecal bile acids (BA) are reduced in *Fxr*^*−/−*^ mice. Bile acids were measured in feces from pregnant (D18) and non-pregnant (D0) wild type (WT) and *Fxr*^*−/−*^ mice. **a** mRNA expression of *Fgf15* in the distal ileum. D0 WT, n = 10; D18 WT, n = 6; D0 *Fxr*^*−/−*^, n = 7, D18 *Fxr*^*−/−*^, n = 8. **b** Total bile acids. **c** Total primary bile acids. **d** Total secondary bile acids. **e** Individual bile acid species. D0 WT, n = 6; D18 WT, n = 8; D0 *Fxr*^*−/−*^, n = 7, D18 *Fxr*^*−/−*^, n = 8. *p* < 0.05 for the following comparisons: ^a^ vs D0 WT, ^b^ vs D18 WT, ^c^ vs D0 *Fxr*^*−/−*^.

Due to the key role of FXR in bile acid homeostasis, it is likely that the composition of bile acids in the intestine and feces is altered in pregnancy and ICP. Gene expression analysis confirmed that ileal *Fgf15* expression is reduced in wild type mice at D18, and suppressed in *Fxr*^*−/−*^ mice (Fig. 4a). Analysis of fecal bile acids showed that pregnant control mice had significantly higher total bile acids in the feces compared to non-pregnant mice (Fig. 4b), due to an increase in both primary and secondary bile acids (Fig. 4c, d). There was a marked reduction in secondary bile acids in D0 *Fxr*^*−/−*^ mice, and both primary and secondary bile acids in D18 *Fxr*^*−/−*^ mice (Fig. 4c, d). Of note, lithocholic acid (LCA) and deoxycholic acid (DCA), the main ligands for TGR5, were significantly reduced in D18 *Fxr*^*−/−*^ mice compared to D18 control mice (Fig. 4e).

## *Tgr5* deficiency impairs glucose tolerance and insulin secretion during pregnancy

**Figure 5:**
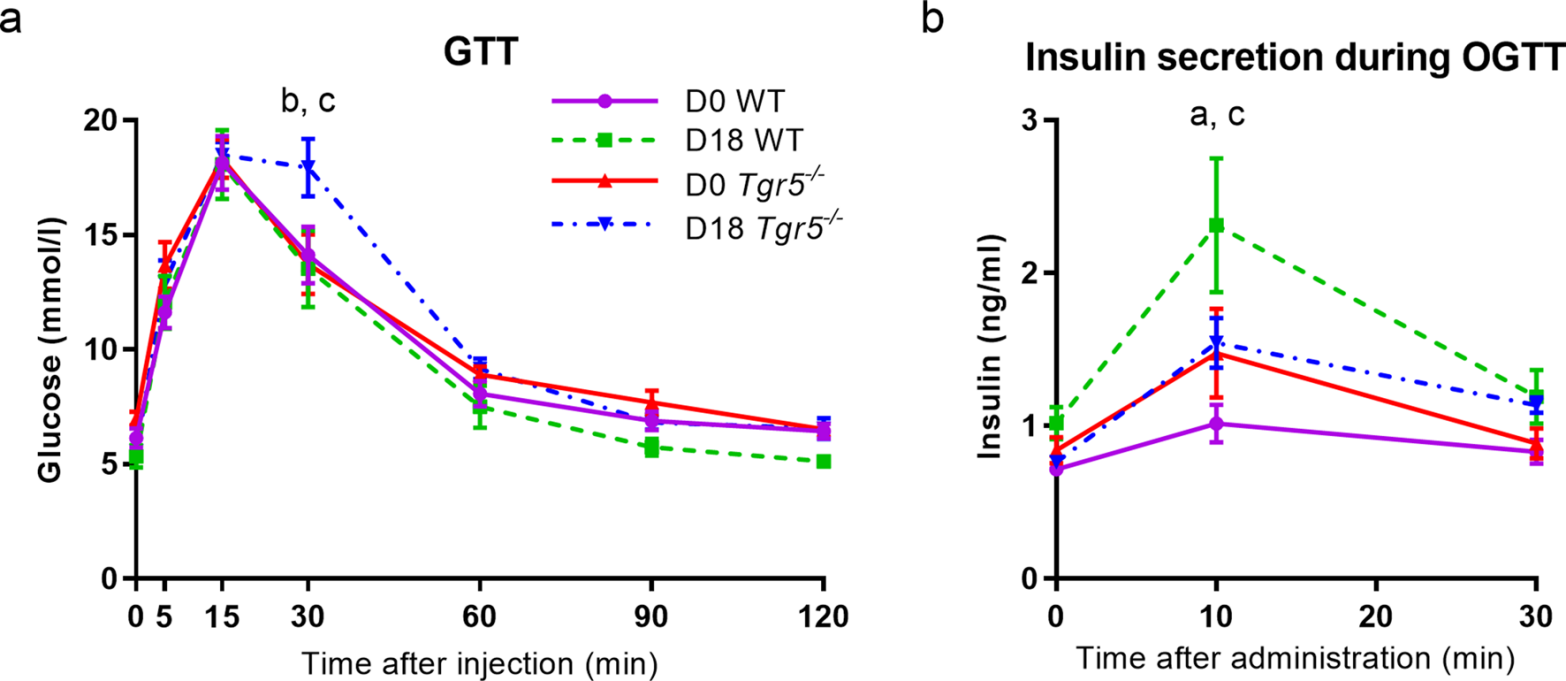
*Tgr5* deficiency impairs glucose tolerance and insulin secretion during pregnancy. Glucose metabolism was assessed in *Tgr5*^*−/−*^ mice and wild type (WT) littermate controls. **a** Glucose tolerance tests (GTT) were performed in non-pregnant (D0) and pregnant (D18) mice. D0 WT, n = 6; D18 WT, n = 5; D0 *Tgr5*^*−/−*^, n = 7; D18 *Tgr5*^*−/−*^, n = 8. **b** Plasma insulin concentrations after oral glucose tolerance test (OGTT; 2 g/kg body weight). D0 WT, n = 4; D18 WT, n = 5; D0 *Tgr5*^*−/−*^, n = 4; D18 *Tgr5*^*−/−*^, n = 9. *p* < 0.05 as determined by repeated-measures two-way ANOVA followed by Bonferroni’s post-hoc test, for the following comparisons: ^a^ D0 WT vs D18 WT, ^b^ D0 *Tgr5*^*−/−*^ vs D18 *Tgr5*^*−/−*^, ^c^ D18 WT vs D18 *Tgr5*^*−/−*^.

Next we investigated the effect of ablation of TGR5 on glucose homeostasis in pregnancy. Pregnant *Tgr5*^*−/−*^ mice were significantly glucose intolerant compared to both D0 *Tgr5*^*−/−*^ and D18 littermate control (WT) mice (Fig. 5a). Insulin secretion in response to oral glucose challenge, to better assess the contribution of TGR5-mediated signalling in the intestine, was also significantly compromised in pregnancy compared to that of D18 WT mice (Fig. 5b). β-cell proliferation was increased in pregnancy in both WT and *Tgr5*^*−/−*^, accompanied by increased islet area at D18, but there were no significant differences between the transgenic and WT mice (Supplementary Table S3).

## Discussion

Women with ICP, a condition characterised by raised serum bile acids, are at increased risk of GDM and have impaired glucose tolerance^6–9^. Given the involvement of the bile acid receptors FXR and TGR5 in glucose homeostasis, we aimed to evaluate the role of these receptors in glucose homeostasis in pregnancy. Our mouse data suggest that both FXR and TGR5 contribute to impaired glucose tolerance in gestation. Administration of CA to pregnant mice disrupts islet expansion and reduces β-cell proliferation, but hypercholanaemia alone does not cause glucose intolerance. Ablation of *Fxr* or *Tgr5* results in glucose intolerance and diminished insulin secretion in pregnant mice. Furthermore, *Fxr*^*−/−*^ mice also have significant insulin resistance. However, ablation of either bile acid receptor was not sufficient to induce the changes in islet phenotype observed in hypercholanaemic mice, and therefore the changes in glucose homeostasis observed in ICP women are likely due to a combination of diminished bile acid receptor activity together with the effect of raised serum bile acids.

In CA-fed pregnant mice, islet size and β-cell proliferation are significantly reduced at D15, when β-cell proliferation is at its peak^37^, while apoptosis is increased. Despite the altered islet phenotype, CA-fed mice are able to maintain insulin secretion to meet the increased demands of pregnancy. This also suggests that CA-feeding does not impact hepatic insulin sensitivity. Our in vitro data confirms previous reports of bile acids inducing glucose-stimulated insulin secretion from islets, and therefore the raised circulating bile acids may enhance insulin secretion, compensating for the reduction in β-cell number.

While the CA-feeding model mimics the increased circulating bile acids seen in ICP, intestinal bile acid levels will also be high since the CA is being ingested. This is unlikely to resemble the intestinal environment in women with ICP as it follows that the reduction in hepatic FXR activity would decrease the export of bile acids into bile. Indeed, we have previously shown that bile acid overload in the intestine of CA-fed mice increases FXR activity in the gut-liver axis, evidenced by repression of hepatic expression of *Cyp7a1*^29^. Intestinal FXR signalling has been shown to be reduced in mouse and human pregnancy^30,31,39^. FXR activity is thought to be reduced further in ICP due to the increase in circulating sulphated progesterone metabolites which are partial agonists of FXR^22^. Therefore, to better understand the impact of suppressed FXR activity on susceptibility to GDM, pregnant *Fxr*^*−/−*^ mice were studied.

Pregnant *Fxr*^*−/−*^ mice displayed significant glucose intolerance and insulin resistance, as well as diminished secretion of insulin, compared to wild type pregnant mice. In support of this, hepatic Akt phosphorylation was reduced, indicative of defective insulin signalling. Of note, while non-pregnant *Fxr*^*−/−*^ mice have increased serum bile acid levels^11,32,40^, there is no further increase in serum bile acids associated with pregnancy, in contrast to wild type pregnant mice^32^, and therefore this could be why there was no evident difference in islet size or β-cell proliferation between wild type and *Fxr*^*−/−*^ mice.

Bile acid concentrations in the feces differed greatly between wild type and *Fxr*^*−/−*^ mice. Despite the assumption that serum bile acid concentrations would be at a similar level to pregnant wild type mice based on our previous study^32^, fecal bile acids were reduced in *Fxr*^*−/−*^ mice, suggesting a reduction in bile acids passing through the intestine. FXR activity can regulate bacterial growth^41^, and in turn, gut microbiota can modify FXR signalling^42^. Indeed, we have recently demonstrated that the gut microbiota changes in pregnancy, with enhanced *Bacteroidetes*-mediated deconjugation of bile acids and reduced uptake of bile acids at the terminal ileum, resulting in impaired enterohepatic feedback^31^. In the present study, we found significantly reduced concentrations of secondary bile acids LCA and DCA, natural ligands for TGR5, in pregnant *Fxr*^*−/−*^ mice. Fecal bile acids more closely reflect the colon than the ileum^42^. The colon has a high proportion of TGR5-expressing entero-endocrine L cells, suggesting that changes in bile acid composition as a result of reduced FXR activity could influence TGR5 activity. Furthermore, progesterone metabolites known to be raised in ICP can also impact TGR5 signalling^24^.

In light of this, and the role of TGR5 in glucose metabolism, we hypothesised that impaired activity of this receptor contributes to glucose homeostasis in ICP, and so pregnant *Tgr5*^*−/−*^ mice were also studied. To our knowledge, glucose homeostasis in pregnant *Tgr5*^*−/−*^ mice has not previously been investigated, however it has been observed in male *Tgr5*^*−/−*^ mice that there was no impact on insulin action and hyperinsulinemic-euglycemic clamp studies revealed that glucose infusion rates were no different to wild type mice^43^. Our data showed that pregnant *Tgr5*^*−/−*^ mice were significantly glucose intolerant and had reduced insulin secretion in response to an oral glucose challenge. As with *Fxr*^*−/−*^ mice there was no impact on islet size or proliferation, which could be due to absence of hypercholanaemia in these mice as previous studies have shown either reduced bile acid pool size^44,45^ or no change in serum bile acid levels in mice lacking *Tgr5*^46^. A limitation of this study is that it was not possible to assess GLP-1 secretion using a fasting-refeeding protocol due to the severe impact of prolonged fasting upon pregnant mice. However, GLP-1 secretion has been previously reported to be reduced in *Tgr5*^*−/−*^ mice^47^. Furthermore, women with ICP have significantly reduced serum concentrations of GLP-1 in response to an oral glucose tolerance test compared with control pregnant women^7^.

Recent studies have confirmed that there is crosstalk between FXR and TGR5. Post-prandial GLP-1 secretion is reduced in *Fxr*^*−/−*^ mice, and the authors describe an FXR responsive element on the *Tgr5* gene promoter^47^. Furthermore, administration of the intestine-specific FXR agonist fexaramine to mice increased serum tauro-LCA and GLP-1, as well as improving insulin and glucose tolerance^48^. This was proposed to be due to increased *Acetatifactor* and *Bacteroides*, key bacteria involved in LCA production from chenodeoxycholic acid and ursodeoxycholic acid. However, it should be noted that *Fxr*^*−/−*^ mice have also been reported to have increased GLP-1 production in response to an oral glucose challenge^49^. Furthermore, inactivation of FXR in the intestine by bile acid sequestrants improves glucose metabolism in obese mice^49^, thought to be due to increased GLP-1 secretion. These contrasting studies highlight the complexity of the relationship between FXR and TGR5, and the need for further research.

Future studies investigating gestational glucose metabolism in tissue-specific FXR knockout mice could shed light on the relative importance of hepatic and intestinal FXR signalling in pregnancy and ICP. It would also be of value to examine the metabolic phenotype of pregnant FXR or TGR5 heterozygous mice, where a partial loss of FXR and TGR5 function may better reflect ICP. Consistent with this, some studies of male *Fxr*^+*/-*^ mice demonstrated insulin resistance and dyslipidaemia^50^ and female *Tgr5*^+*/-*^ mice have been reported to have increased fat mass compared to WT mice, although this was not statistically significant^44^. Prospective clinical studies are also needed to more closely examine the temporal relationship between bile acid and glucose metabolism in pregnancy.

The data presented herein demonstrate that a hypercholanaemic environment in pregnancy impacts islet morphology, and that pregnant mice lacking FXR and TGR5 have impaired glucose homeostasis. Therefore, it is likely that a combination of reduced activity of both bile acid receptors FXR and TGR5, together with raised circulating bile acid levels, contributes to the altered glucose metabolism in pregnant women with ICP.

## Supplementary information

Supplementary Information.

## Data Availability

The datasets generated during and/or analysed during the current study are available from the corresponding author on reasonable request.
