# Supplementary material for: Maternal glucose homeostasis is impaired in mouse models of gestational cholestasis
Source: Sci Rep. 2020 Jul 13;10:11523. doi: 10.1038/s41598-020-67968-6 (PMC7359298; doi:10.1038/s41598-020-67968-6)
Supplement: Supplementary file 1 — Supplementary Information. [file 41598_2020_67968_MOESM1_ESM.docx]

**Maternal glucose homeostasis is impaired in mouse models of gestational cholestasis**

**Online Supplementary Material**

Elena Bellafante, Saraid McIlvride, Vanya Nikolova, Hei Man Fan, Luiza Borges Manna, Jenny Chambers, Mavis Machirori, Anita Banerjee, Kevin Murphy, Marcus Martineau, Kristina Schoonjans, Hanns-Ulrich Marschall, Peter Jones and Catherine Williamson

Supplementary Methods

*Glucose-stimulated insulin secretion (GSIS) in murine islets*

8-10 week old male C57BL/6J mice were purchased from Charles River UK Ltd. All experiments were performed in accordance with the Animals (Scientific Procedures) Act 1986 Amendment Regulations 2012 and approved by King’s College London’s Animal Welfare and Ethical Review Body. Mice were euthanised by cervical dislocation and the pancreas was inflated with collagenase (1 mg/ml), dissected, and islets of Langerhans isolated as previously described ^1^. Islets were incubated overnight in RPMI media (10% FCS, 1% P/S) at 37°C, 5% CO_2_.

For GSIS experiments, islets were incubated in Gey & Gey buffer, containing 2 mM or 20 mM glucose. Islets were pre-treated with 2mM glucose for 1 hour at 37 °C, 5% CO_2_, then aliquoted into Eppendorf tubes (5 islets per tube, 8 – 10 replicates per condition) containing Gey & Gey buffer at 2 and 20 mM glucose, with 50 μM taurocholic acid (TCA) or 50 μM taurochenodeoxycholic acid (TCDCA) and incubated in a water bath at 37°C. After 1 hour, the samples were placed on ice and the supernatant collected and stored at -20°C until analysis. Insulin content of incubation medium was measured by in-house radioimmunoassay, as previously described ^2^.

References

1. Bowe, J.E. *et al.* Kisspeptin stimulation of insulin secretion: mechanisms of action in mouse islets and rats. *Diabetologia* **52**, 855-862 (2009).

2. Jones, P.M., Salmon, D.M. & Howell, S.L. Protein phosphorylation in electrically permeabilized islets of Langerhans. Effects of Ca2+, cyclic AMP, a phorbol ester and noradrenaline. *Biochem. J.* **254**, 397-403 (1988).

Supplementary Data


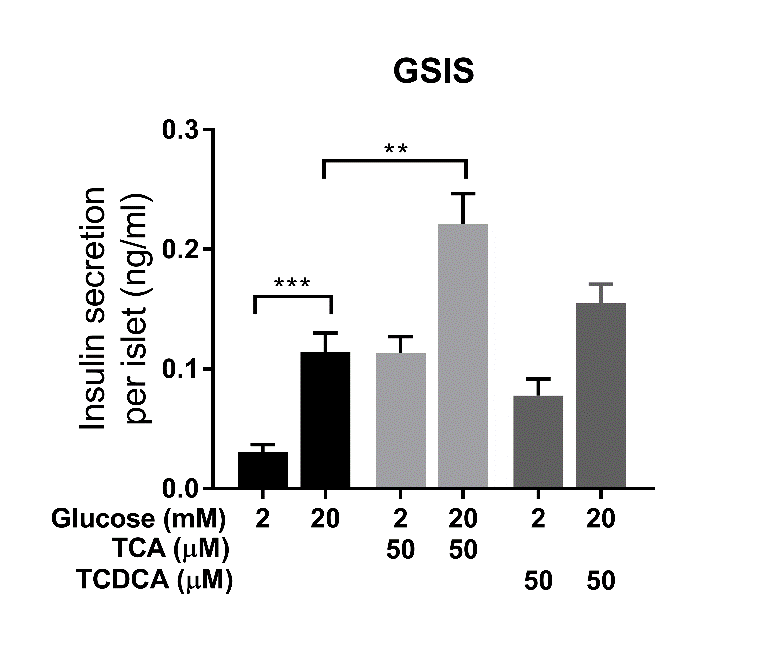


**Supplementary Figure S1 Glucose stimulated insulin secretion (GSIS)**

Static incubation of murine islets with glucose and taurocholic acid (TCA) or taurochenodeoxycholic acid (TCDCA) (n = 6). Data presented as mean ± SEM. **p < 0.01, ***p < 0.001, analysis determined by Kruskal-Wallis test, followed by Dunn's post hoc test.

**Supplementary Table S1**. Percentage BrdU-positive cells in islets from WT and Fxr^-/-^ mice

|  | Mean % BrdU+ cells (±SEM) |
| --- | --- |
| D0 WT (n = 10) | 9.5 (1.82) |
| D0 Fxr^-/-^ (n = 4) | 1.1 (1.14) |
| D15 WT (n = 35) | 41.0 (1.14) * |
| D15 Fxr^-/-^ (n = 27) | 33.8 (3.14) ^#^ |
| D18 WT (n = 12) | 34.4 (2.20) * |
| D18 Fxr^-/-^ (n = 61) | 33.8 (1.80) ^#^ |

*****p < 0.05 vs D0 WT, ^#^p < 0.05 vs D0 Fxr^-/-^, as determined by one-way ANOVA followed by Newman-Keuls multiple comparison test

**Supplementary Table S2.** Mean islet area in WT and Fxr^-/-^ mice

|  | Mean islet area (±SEM)  mm^2^ |
| --- | --- |
| D0 WT (n = 17) | 6.997 (1.23) |
| D0 Fxr^-/-^ (n = 4) | 12.77 (10.42) |
| D15 WT (n = 24) | 13.04 (3.15) |
| D15 Fxr^-/-^ (n = 27) | 15.32 (2.85) |
| D18 WT (n = 31) | 8.91 (1.23) |
| D18 Fxr^-/-^ (n = 61) | 11.34 (1.30) |

No significant differences, analysis determined by Kruskal-Wallis analysis, followed by Dunn's post hoc test.


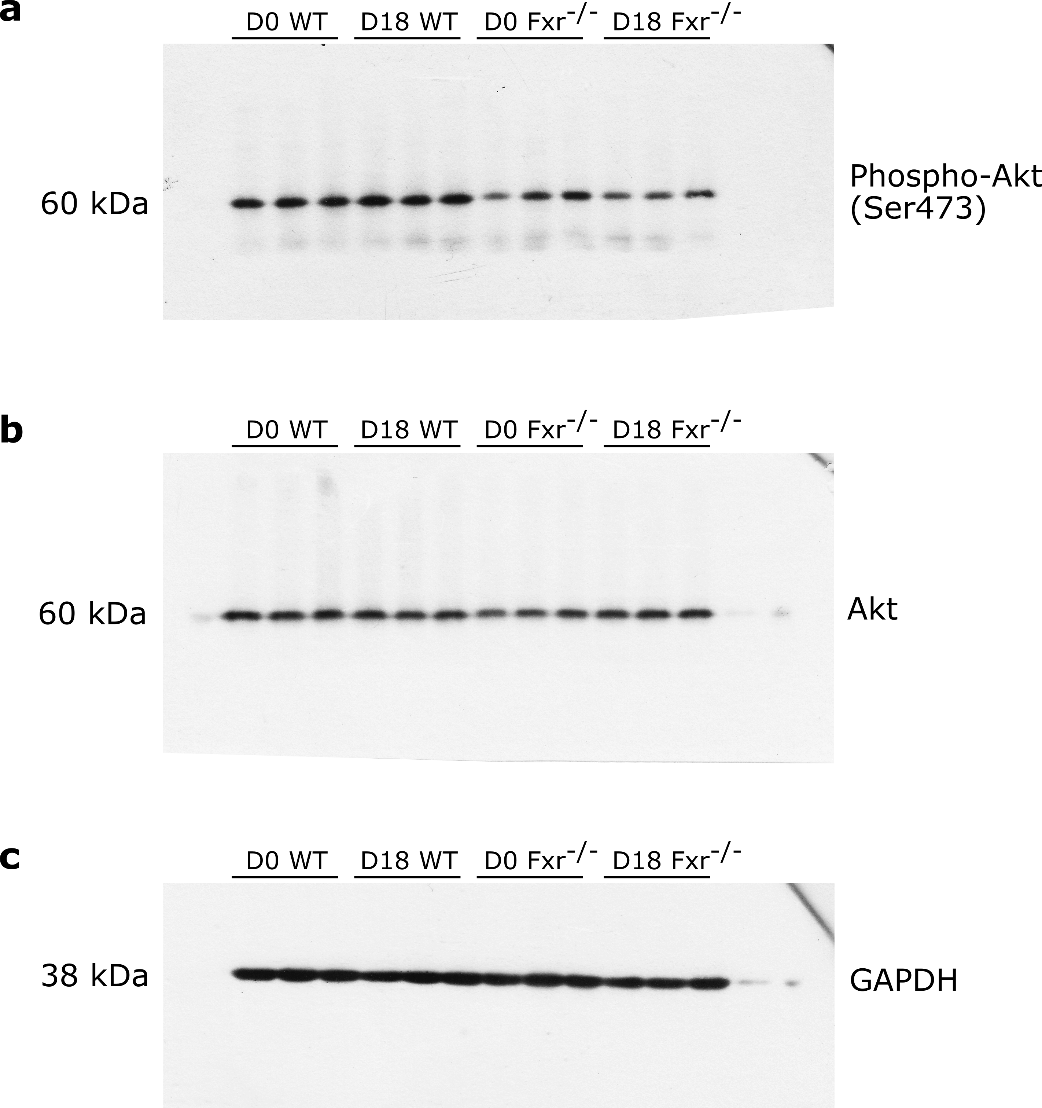


**Supplementary Figure S2**

Western blot analysis of hepatic (a) phospho-Akt (Ser473), (b) total Akt and (c) GAPDH in non-pregnant and pregnant wild type (WT) and *Fxr^-/-^* mice.

**Supplementary Table S3.** Percentage of BrdU-positive cells in islets from WT and Tgr5^-/-^ mice

|  | Mean % BrdU+ cells (±SEM) | Mean islet area (±SEM)  mm^2^ |
| --- | --- | --- |
| D0 WT (n = 51) | 15.81 (2.05) | 16.61 (2.44) |
| D0 Tgr5^-/-^ (n = 62) | 8.546 (0.85) * | 17.55 (2.1) |
| D15 WT (n = 56) | 27.4 (2.23) * | 17.89 (2.98) |
| D15 Tgr5^-/-^ (n = 67) | 24.55 (1.97) ^#^ | 10.45 (1.24) |
| D18 WT (n = 81) | 25.23 (1.44) * | 29 (3.31) ^$^ |
| D18 Tgr5^-/-^ (n = 55) | 26.66 (1.93) ^#^ | 26.23 (4.47) ^‡^ |

*****p < 0.05 vs D0 WT, ^#^p < 0.05 vs D0 Tgr5^-/-^, as determined by one-way ANOVA followed by Newman-Keuls multiple comparison test. ^$^p < 0.05 vs D15 WT, ^‡^p < 0.05 vs D15 Tgr5^-/-^, as determined by Kruskal-Wallis analysis, followed by Dunn's post hoc test.
